# A content analysis of Australian television advertising: focus on child and adolescent oral health

**DOI:** 10.1186/s12887-018-1356-8

**Published:** 2018-12-07

**Authors:** Amit Arora, Caroline M. Bowman, Stephanie J. P. Chow, Jack Thepsourinthone, Sameer Bhole, Narendar Manohar

**Affiliations:** 1 School of Science and Health, Western Sydney University, Campbelltown Campus, Locked Bag 1797, Penrith, NSW 2751 Australia; 2 Translational Health Research Institute, Western Sydney University, Campbelltown Campus, Locked Bag 1797, Penrith, NSW 2751 Australia; 3 Discipline of Child and Adolescent Health, Sydney Medical School, Faculty of Medicine and Health, The University of Sydney, Westmead, NSW 2145 Australia; 4 Oral Health Services, Sydney Local Health District and Sydney Dental Hospital, NSW Health, Surry Hills, NSW 2010 Australia; 5 Sydney Dental School, Faculty of Medicine and Health, The University of Sydney, Surry Hills, NSW 2010 Australia

**Keywords:** Dental caries, Television advertisement, Australia, Cariogenic, Content analysis

## Abstract

**Background:**

Children’s preferences for cariogenic foods and/or drinks has been proven to be associated with exposure to advertisements. This study aimed to assess and compare the proportion of cariogenic food and /or drink advertisements aired on three metropolitan Sydney commercial television channels at different broadcast times during school term and school holidays.

**Methods:**

Three Sydney free-to-air television channels (Channels Seven, Nine, and Ten) were recorded between June 2016 and January 2017. Two weekdays and one weekend day were recorded for a week for each channel during the school term and school holidays, respectively. All channels were recorded from 0630 h until 2300 h. Food and/or drink advertisements were categorised according to the time they were aired and their sugar and acid content. For each channel, school holiday data was compared with school term data. Pearson chi-squared testing was used to determine the difference in advertisements rates across TV channels and broadcast times including school holidays and school term.

**Results:**

The proportion of food and/or drink advertisements for all networks was less than 10% of all advertisements. Overall, Channel Ten had the most food and/or drink advertisements (39.74%) and Channel Seven had the lowest (28.60%). Channel Ten aired the largest proportion of food and/or drink advertisements (27.18%) during school term Channel Nine aired the highest number of food and/or drink adverts (15.50%) during school holidays. There were more food and/or drink advertisements during children’s viewing hours compared to overlap, adult, and other viewing periods respectively, with Channel Ten airing the highest advertisements (15.72%) and Channel Seven airing the least (11.35%) food and/or drink advertisements. For all analyses, Pearson chi-square tests had a *p*-value < 0.001.

**Conclusion:**

Although the overall proportion of food and/or drink advertisements aired on Sydney television is low, the advertisements containing high sugar and /or acid were broadcasted more during children’s viewing times than other times and during school term compared to school holidays.

## Background

Dental caries (tooth decay) is recognised as a global public health concern by the World Health Organization (WHO) [1]. The Global Burden of Disease 2015 Study [2] reports that nearly 573 million children are affected by untreated dental caries in primary (baby) teeth. Further, untreated caries in permanent (adult) teeth was the most prevalent condition in all of GBD 2015 affecting 2.5 billion people worldwide [2]. In Australia, recent evidence shows that caries rates in the primary dentition has been increasing since 1995 [3] and is considered to be a strong predictor of caries in the permanent dentition [4] and has the potential to produce significant costs to the health sector [5]. Where hospitalisation is necessary, management of this entirely preventable disease is estimated to directly cost US$3300 (over $4000 AUD) per case without accounting for the social and economic costs to the family [5]. Although dental caries is a multifactorial disease [6], detrimental changes in diet, in particular, increased frequency of snacking on sweet foods and increased consumption of sugar sweetened beverages have been majorly attributed to the rise in caries incidence [7–10].

The consumption of cariogenic foods is influenced by children’s television advertising and marketing [11]. Children’s preferences for foods and drinks has been proven to be associated with exposure to television advertisements [12–15]. Previous observational research has noted that high levels of television viewing are associated with greater consumption of energy-dense, nutrient-poor food and drinks [13–17]. More recent evidence, however; suggests it may be the advertising, rather than the television viewing per se, that is particularly detrimental [18]. The use of persuasive marketing techniques such as employing celebrities, cartoon characters, athletes, and promotional gifts associated with advertising are commonly used in the marketing of unhealthy/non-core foods and drinks for children [19, 20]. Such persuasive marketing is proven to promote brand recognition, food preferences, purchase requests and food consumption in children [13–15].

Internationally, there is a dearth of evidence on television advertising and oral health. A study by Sukumaran et al. [21] analysed the content of advertisements in India and reported that 55.6% adverts were on food, of which 46.8% focused on sugar-rich foods. On the other hand, Al-Mazyad et al. [22] reported that nearly two-thirds of the food adverts in UK were for items potentially harmful to oral health. Another study by Morgan and colleagues [23] in the UK reported that 16.4% of the advertisements time was devoted to food products, and 6.3% of all advertising time to potentially cariogenic products. Rodd and Patel [24] also reported that 34.8% adverts on UK television were related to food and drinks, of which 95.3% were for food and drinks high in sugar and/or acid. A recent systematic review and a meta-analysis [25] that assessed the content of television advertising in terms of oral health concluded that 38% of the advertisements were related to food and of those, 70.6% were related to cariogenic foods in particular. Although the meta-analysis is recent [25], some international literature was not included in the meta-analysis [23, 26], and that to the best our knowledge there seems to be no Australian research on television advertising and oral health.

In Australia, the current system of regulating food advertising aimed at children comprises of both mandatory and self-regulatory elements. The mandatory element is embedded in Children’s Television Standards, which cover television viewing times for children, and is regulated by the Australian Communications and Media Authority [27]. The mandatory Children’s Television Standards does not restrict the promotion of advertisements in general; however, they do restrict promotions involving celebrities for children’s programs [27]. Therefore, it is recommended that food advertisements should be restricted before, during, and after, all the television programs aimed specifically for children [28, 29]. Furthermore, two self-regulatory codes, Responsible Children’s Marketing Initiative [30] and the Australian Quick Service Restaurant Industry Initiative [31], have been introduced in Australia, aimed at reducing children’s exposure to advertisements promoting unhealthy foods and drinks. Despite these mandatory and self-regulatory guidelines, a review of the literature has shown that unhealthy foods and drink items are still often advertised on local television [32–36].

Content analyses of children’s food advertising focusing on unhealthy/non-core foods have concluded that, despite regulatory changes, Australian children continue to see a large amount of advertising for non-core foods [28, 29, 32–36]. This is concerning given that the 2011–12 Australian Health Survey highlighted that children and young people (5–17 years) spent almost 136 min per day in sedentary activities such as television viewing [37]. Although there is some research on Australian television advertising in relation to obesity [28, 29, 32–36], there is paucity of data about television advertisements related to oral health [38]. This research addresses this gap by examining the content of television advertising across three most common free-to-air Sydney channels with respect to oral health.

The aims of this study are:

1. To examine the distribution and content of television advertising across three metropolitan Sydney free-to-air channels with a specific reference to oral health.
2. To compare the proportion of food and/or drink advertisements aired on three metropolitan Sydney free-to-air television channels during school holidays and school term, respectively, with a focus on oral health.
3. To compare the proportion of food and/or drink advertisements aired on three metropolitan Sydney free-to-air television channels based on the broadcast time with a focus on oral health.

## Methods

Three most commonly viewed free-to-air Sydney television channels (Channels Seven, Nine, and Ten) were recorded between May 2016 and October 2016. A total of 6 days i.e., two weekdays and one weekend day, during school term and school holidays respectively. The channels and days were selected based on television ratings data obtained from OzTAM (Australian Television Audience Measurement) (available at http://www.oztam.com.au) and based on prior Australian research [32, 39]. Public holidays and days having large scale sporting events were excluded to ensure that the data represented typical and/or routine broadcasting. All channels were recorded from 0630 h until 2300 h. The advertisements of all the three selected channels were recorded simultaneously onto the hard-discs or DVD’s.

### Coding

All advertisements were viewed and analysed by three researchers (SC, CB, and AA). Advertisements were initially categorised as “food and/or drink advertisements” or “non-food and/or drink advertisements”. Subsequently, the “food and/or drink advertisements” were sub-categorised based on two main criteria:

1. *Broadcast time:* Broadcast time was divided into four categories. Table 1 shows the time periods of each broadcast time:

- Peak child-viewing time, also known as C band time-period (as defined in Children’s Television Standards [27]).
- Peak adult-viewing time (determined as per Australian television networks [40])
- Overlap time-period (between 1600 h and 2030 h - when children watch television under adult supervision).
- Other time period

2. *Food and/or drink type:* These categories are based on sugar and acid content of the food and/or drink as described by Rodd and Patel [24]. The categories are:

- Group 1 represents foods and/or drinks high in sugar, such as confectionery (sweets, biscuits, cakes), breakfast cereals with added sugar, breakfast bar and flavoured milk products.
- Group 2 represents foods and/or drinks with high acid content, such as sugar-free soft drinks.
- Group 3 represents foods and/or drinks with high sugar and acid content, including soft drinks (carbonated and non-carbonated).
- Group 4 represents foods and/or drinks with low sugar and low acid content. This includes dairy products, breakfast cereals with no added sugar, tea/coffee and convenience food.

**Table 1:** Summary of time periods/brackets for weekdays and weekends during School Term and School Holidays

	School Term		School Holiday	
Broadcast time	Weekdays	Weekend	Weekdays	Weekend
Child	0700-0830 1600-2030	0700-2030	0700-2030	0700-2030
Adult/prime time	2030-2300	1800-2300	2030-2300	1800-2300
Overlap	1800-2030	1800-2030	1800-2030	1800-2030
Other	0630-0700 0830-1600	0630-0700	0630-0700	0630-0700

Only those advertisements which were aired during the in-between breaks of televised programs were considered for the study purpose, whereas ‘*infomercials’* during the televised programs or advertisement banners displayed at the corner of televised programs were not included. Duration of advertisements or frequency of specific adverts were not recorded. Adverts with multiple products (e.g., weight loss programs) in which food and/or drink was just one of the advertised products, were classified as “non-food and/or drink” advertisements, since they were not designed to actively promote a specific food and/or drink product. Additionally, advertisements promoting alcohol were classified as “non-food and drink advertisement” since they were not targeted towards children.

### Statistical analysis

Statistical Package for Social Science (SPSS) version 22 (SPSS for Windows, SPSS Inc., Chicago, IL, USA) was used for data management and analysis. Data were analysed descriptively to determine the proportion of food and/or drink advertisements according to sugar and acid content and viewing time-periods across each TV channel. Additionally, using a Bonferonni adjusted *α* of 0.01, a series of one-way chi-square analyses were conducted to compare the proportion of advertisements across TV channels; during school term and school holidays, and during peak child, overlap, adult, and other viewing times, respectively.

## Results

**Fig. 1:**
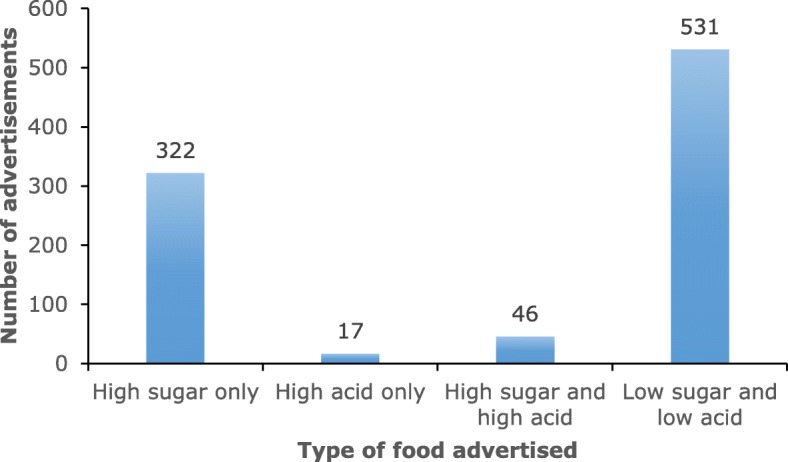
Distribution of cariogenic food and/or drinks advertisements categorised according to nutritional content

A total of 297 h of television programs including advertisements was recorded. There were 12,121 advertisements aired during the 6 days of recording. Food and/or drink advertisements as a proportion of all advertisements aired during the six-day study period was less than 10% for all TV networks. Of all advertisements, 916 (7.56%) were of food and/or drinks while 11,205 (92.44%) advertisements were non-food and/or drink related. Figure 1 shows the distribution of advertisements across the four food and/or drink categories. Of the 916 food and/or drink advertisements, 5.02% were for high sugar and high acid foods and/or drinks, 35.15% were for high sugar foods and/or drinks, 57.96% for foods and/or drinks that were low in both sugar and acid, while 1.86% were for food and/or drink items high in acid content only. The one-way chi-square test revealed a significant difference in the proportion of advertisements across the four food and/or drink categories, *Χ*^2^ = 778.54, *p* < .001.

Table 2 shows the distribution of food and/or drink advertisements across all networks. A significant difference in the number of food and/or drink adverts across all recorded networks was observed, *Χ*^2^ = 18.19, *p* < .001. Overall, Channel Ten aired the highest number (*n* = 364) of food and/or drink advertisements over the six-day study period while Channel Seven aired the lowest number (*n* = 262) of food and/or drink adverts.

**Table 2:** Distribution of food and/or drink advertisements according to broadcast channels^*^

Television Channel	Seven		Nine		Ten	
	*n*	(%)	*n*	(%)	*n*	(%)
High Sugar Group	97	10.59	95	10.37	130	14.19
Breakfast cereals with added sugar	7	0.76	6	0.66	10	1.09
Confectionery (sweets, biscuits, cakes) and snacks	85	9.28	74	8.08	112	12.23
Flavoured milk drinks	5	0.55	15	1.64	8	0.87
High acid group	7	0.76	1	0.11	9	0.98
Sugar-free soft drinks and fruit juices	7	0.76	1	0.11	9	0.98
High sugar and high acid group	17	1.86	10	1.09	19	2.07
Sugar-sweetened drinks	17	1.86	10	1.09	19	2.07
Low sugar and low acid group	141	15.39	184	20.09	206	22.49
Dairy products	13	1.42	8	0.87	10	1.09
Breakfast cereals with no added sugar	5	0.55	11	1.20	14	1.53
Tea/coffee	16	1.75	4	0.44	13	1.42
Convenience foods	107	11.68	161	17.58	169	18.45
Total food advertisements	262	28.60	290	31.66	364	39.74

^*^Chi-square (*Χ*^2^) = 18.19, *p* < .001

Table 3 shows the percentage of food and/or drink advertisements (sub-categorised into four food and/or drink groups) across all three channels according to whether they were aired during school term or school holidays. Channel Ten aired the largest proportion of food and/or drink advertisements (*n* = 249) during school term Channel Nine aired the highest number of food and/or drink adverts (*n* = 142) during school holidays. Overall, Channel Seven aired the lowest percentage of food and/or drink related advertisements during both school term (*n* = 147) and school holidays (*n* = 115). A one-way chi-square revealed a statistically significant difference between the proportion of food and/or drink adverts during school term and school holidays, *Χ*^2^ = 32.30, *p* < .001, whereby food and/or drink advertisements were more frequently aired during the school term.

**Table 3:** Distribution (%) of food advertisements according to school term (ST) and school holiday (SH) broadcast periods^*^

Television Channel	Seven		Nine		Ten	
	SH	ST	SH	ST	SH	ST
High sugar group	4.48	6.11	3.60	6.77	2.51	11.68
Breakfast cereals with added sugar	0.55	0.22	0.00	0.66	0.66	0.44
Confectionary (sweets, biscuits, cakes) and snacks	3.38	5.90	2.73	5.35	1.86	10.37
Flavoured milk drinks	0.55	0.00	0.87	0.76	0.00	0.87
High acid group	0.00	0.76	0.11	0.00	0.33	0.66
Sugar-free soft drinks and fruit juices	0.00	0.76	0.11	0.00	0.33	0.66
High sugar and high acid group	0.87	0.98	0.87	0.22	0.22	1.86
Sugar-sweetened drinks	0.87	0.98	0.87	0.22	0.22	1.86
Low sugar and low acid group	7.21	8.19	10.92	9.17	9.50	12.99
Dairy products	0.66	0.76	0.00	0.87	0.00	1.09
Breakfast cereals with no added sugar	0.22	0.33	0.76	0.44	0.33	1.20
Tea/coffee	0.76	0.98	0.00	0.44	0.00	1.42
Convenience foods	5.57	6.11	10.15	7.42	9.17	9.28
Total	12.55	16.05	15.50	16.16	12.55	27.18

^*^Chi-square (*Χ*^2^) = 32.30, *p* < .001

Table 4 shows the percentage of food and/or drink advertisements (sub-categorised into four food and/or drink groups) across all three channels according to the broadcast time i.e. whether they were aired during peak child, overlap, adult, and other viewing periods. When comparing the proportion of overall food and/or drink advertisements distributed across child, adult, overlap, and other viewing periods; it was revealed that there is a wide range of distribution i.e., from a minimum value of 3.49% to a maximum value of 15.72%. A statistically significant difference was found between the proportion of food and/or drink adverts aired during the four specified viewing periods, *Χ*^2^ = 118.75, *p* < .001 irrespective of the TV networks. Foods and/or drinks were advertised more frequently during the peak child-viewing period (*n* = 359) compared to the overlap (*n* = 216), adult (*n* = 211), and other (*n* = 130) viewing periods. Furthermore, a statistically significant difference was revealed between the proportion of food and/or drink adverts on the three broadcast channels across other viewing times (*Χ*^2^ = 13.49, *p* < .001), respectively. Channel Ten had the highest proportion of food and/or drink advertisements during child (*n* = 144), overlap (*n* = 83), adult (*n* = 74), and other viewing periods (*n* = 63, 6.48%). Conversely, Channel Seven had the lowest proportion of food and/or drink advertisements for child (*n* = 104), overlap (*n* = 57), adult (*n* = 66), but not other viewing periods (n = 35).

**Table 4:** Distribution (%) of food advertisements according to the viewing times

Television Channel	Seven				Nine				Ten			
Time slot	C	O	A	O*	C	O	A	o	C	O	A	o
High sugar group	3.71	2.62	3.06	1.20	3.38	2.40	3.06	1.53	6.11	2.84	2.84	2.40
Breakfast cereals with added sugar	0.11	0.33	0.33	0.00	0.11	0.11	0.00	0.44	0.76	0.11	0.00	0.22
Confectionery (sweets, biscuits, cakes) and snacks	3.49	2.29	2.29	1.20	2.51	2.29	2.18	1.09	5.35	2.40	2.29	2.18
Flavoured milk drinks	0.11	0.00	0.00	0.00	0.76	0.00	0.87	0.00	0.00	0.33	0.55	0.00
High acid group	0.33	0.00	0.00	0.44	0.11	0.00	0.00	0.00	0.22	0.33	0.11	0.33
Sugar-free soft drinks and fruit juices	0.33	0.00	0.00	0.44	0.11	0.00	0.00	0.00	0.22	0.33	0.11	0.33
High sugar and high acid group	0.76	0.33	0.55	0.22	0.33	0.33	0.33	0.11	0.76	0.33	0.11	0.87
Soft drinks	0.76	0.33	0.55	0.22	0.33	0.33	0.33	0.11	0.76	0.33	0.11	0.87
Low sugar and low acid group	6.55	3.28	3.60	1.97	8.30	5.57	4.37	1.86	8.62	5.57	5.02	3.28
Dairy products	0.55	0.55	0.33	0.00	0.66	0.11	0.11	0.00	0.11	0.33	0.33	0.33
Breakfast cereals with no added sugar	0.22	0.11	0.22	0.00	0.87	0.22	0.11	0.00	0.55	0.44	0.00	0.55
Tea/coffee	0.76	0.22	0.44	0.33	0.33	0.00	0.00	0.11	0.55	0.00	0.00	0.87
Convenience foods	5.02	2.40	2.62	1.64	6.44	5.24	4.15	1.75	7.42	4.80	4.69	1.53
Total	11.35	6.22	7.21	3.82	12.12	8.30	7.75	3.49	15.72	9.06	8.08	6.88

*C* Child viewing hours, *O* Overlap viewing hours, *A* Adult viewing hours, and *o* Other viewing hours

^*^Chi-square (*Χ*^2^) = 13.49, *p* < .001 for other viewing hours

## Discussion

To the best of our knowledge, the present study seems to be the first Australian research related to content analysis on television advertisements with a focus on oral health. Specifically, the food and/or drink advertisements were studied for specific peak viewing periods across three networks during the school term and school holidays in metropolitan Sydney. The proportion of food and/or drink advertisements for all networks was less than 10% of overall televised transmission time. Channel Ten had the most food and/or drink advertisements and Channel Seven had the lowest proportion of food and/or drink adverts. This proportion is considerably less than that reported by Kelly et al. (25.5%) [19], Rodd and Patel (34.8%) [24], and Hebden et al. (28%) [35] in their respective studies. The Australian study by Hebden et al. [35] reported data for channels specifically targeting children < 12 years, different times of the day (7.00 until 20.30), and different time of the year (February 2009) which may account for differences in findings. Likewise, another Australian study by Kelly and colleagues [32] reported a decreasing trend for food and beverages decreased over the three-year period; from 26% in 2006 and 25% in 2007 to 15% in 2008. It is also worthy to note that that the current Children’s Television Standards [27] which cover television viewing times for children that is regulated by the Australian Communications and Media Authority came into action in 2014 which may also account for lower proportion of food advertisements in our study. However*,* a matter of concern is that the proportion of food and/or drink advertisements were the highest during the child-viewing period, during which it is highly likely that the adverts are viewed by children. Such exposure may have a strong influence on persuading children towards an unhealthy dietary lifestyle.

Although food and/or drink advertisements were low compared to the total adverts aired, the findings of this study highlighted that significant amount of the adverts promoted cariogenic food and/or drink products. Approximately, 40% of food and/or drink advertisements were related to dietary items that were high in either sugar or acid content, or both sugar and acid content. This percentage is less in comparison to an earlier Australian study by Kelly et al. which reported the proportion of food advertisements for high sugar or acid, or high sugar and acid products to be 61.3% [19]. The UK study by Rodd and Patel [24] reported this proportion to be over 55%. These differences are possibly due to variations across countries, collecting a small amount of data over a short period of time, and collecting data during a more restricted period of the day. Nonetheless, food and/or drink advertisements of products containing high sugar and/or high acid pose a detrimental risk to oral health of children. Additionally, the consumption of high sugar foods is of concern for other public health issues such as obesity and diabetes [41–43]. This grants further reason for the government to implement stronger regulations on television advertising of unhealthy foods and/or drinks aimed towards children.

The higher proportion of food and/or drink advertisements during school term is probably in compliance with recommended guidelines [27] prohibiting airing of unhealthy/non-core foods and/or drinks adverts during child-viewing time, especially during school holidays. Amongst all types of food and/or drink items, convenience food adverts predominated both during school term and holidays across all channels. Similarly, excessive advertising for foods and/or drinks potentially detrimental to oral health were also observed by other researchers [22–24, 44, 45], thereby concluding that children are being excessively persuaded towards high sugar products through children’s and primetime television commercials. The present study has focused primarily on the role of sugar and acid content, frequent intake of which has been correlated with dental caries and tooth erosion.

A positive finding drawn from the present study is that the high proportion of food and/or drink advertisements were those promoting non-cariogenic dietary items. This finding is in contrast to findings of similar studies (i.e. children’s television advertising) from different countries [46–48] and may reflect mandatory and self-regulatory advertising regulations*.*

Following the release of revised standards for television food advertising for children by the Australian Communications and Media Authority in 2009, there has been a decrease in the overall rate of food and/or drink advertisements (adverts per hour per channel) [27, 32]. All networks demonstrated a reduction in the proportion of food and/or drink advertisements aired during school holidays compared to school term. Children’s Television Standards [27], Australian Food and Grocery Council self-regulatory initiatives [30, 31, 49], and the Commercial Television Industry Code of Practice [50] have likely been influential in reducing the overall number of food advertisements during child viewing hours; however, children are still exposed to a significant number of food and/or drink advertisements. The proportion of advertisements for non-core foods and/or drinks however, has remained almost steady since 2006 (50% in 2006, 48% in 2007, 49% in 2008) [32].

In 2009, the Australian Food and Grocery Council promulgated a national self-regulatory initiative relating to responsible food marketing for children, encompassing food marketing on subscription services which was also adopted by several food companies [30]. Earlier Australian studies reported a higher rate of non-core food advertisements [35, 45] in comparison to the present study which might, in some way, be an outcome of the above mentioned responsible marketing policies adopted by food companies. However, such self-regulatory policies have limited government regulation and industry self-regulation [45]. Hence, government involvement is required to ensure stronger implementations to further control the promotion of unhealthy foods through television advertisements and ensure that children are persuaded towards healthier food choices—favourable to their oral health and overall growth and development.

The current study provides an insight into the extent of food advertising to children on three popular Australian channels with a particular focus on post-regulation advertising of foods potentially damaging to oral health. Some of the limitations of our study were limited number of channels that were recorded, a short recording period (i.e. 2 week days and one weekend day in school term and school holidays), and only considering the number of advertisements and not whether they were repeated, as some products may be advertised more often. Further, it is also difficult to prove that television advertising has a direct effect on oral health, given the multifactorial nature of dental caries and erosion. It is suggested that future studies be done with longer recording periods, and a broader variety of television channels for generalisability of the findings. Furthermore, other viewing modes such as Netflix, paid cable television, You Tube, and mobile phone applications, should be taken into account when evaluating children’s exposure to food advertisements. Future studies should also account for oral hygiene products particularly fluoride, which have a protective effect towards child and adolescent oral health.

## Conclusion

Although the overall proportion of food and/or drink advertisements aired on Sydney television is low, the advertisements containing high sugar and /or acid were broadcasted more during children’s viewing times than other times and during school term compared to school holidays. Potentially, due to such adverts, there may be higher probability of parents being persuaded to procure unhealthy foods for their children, thereby posing a threat in terms of children’s oral health alongside other health risks such as obesity and diabetes. This calls for stronger government involvement to restrain promotion of unhealthy food and/or drinks to children.

## Abbreviations

(PG): Parental Guidance
(SH): School Holiday
(ST): School Term
(TV): Television
